# Diet screeners and their association with constipation and stool consistency in school-aged children

**DOI:** 10.1590/1984-0462/2026/44/2025276

**Published:** 2026-06-29

**Authors:** Giovana Canela Spadotto, Maria Antonieta de Barros Leite Carvalhaes, Cristina Maria Garcia de Lima Parada, Michelly da Silva Alves, Maria Isabel Cuaio, Camila Maria de Arruda, Débora Avellaneda Penatti, Caroline de Barros Gomes

**Affiliations:** a Universidade Estadual Paulista, Faculdade de Medicina, Botucatu, SP, Brazil.; b Universidade Estadual Paulista, Instituto de Biociências, Botucatu, SP, Brazil.; c Universidade de Marília, Marília, SP, Brazil.; d Centro Universitário de Adamantina, Adamantina, SP, Brazil.

**Keywords:** Food consumption, Constipation, Feces, Child health, Consumo alimentar, Constipação, Fezes, Saúde da criança

## Abstract

**Objective:**

To investigate the association of constipation and stool consistency with diet quality screeners in school-aged children.

**Methods:**

A cross-sectional analysis was conducted using data from the second phase of the Botucatu Infant Cohort Study. During home visits, stool consistency was assessed using the Bristol Stool Form Scale, and the presence of constipation (yes/no) was defined according to the adapted Rome IV criteria. Dietary intake (yes/no) on weekdays and weekends of the seven foods used as dietary quality screeners by the Food and Nutrition Surveillance System (SISVAN) was assessed through telephone interviews. Two scores were calculated: the Healthy Eating Score (three fresh or minimally processed foods, range: 0–6) and the Unhealthy Eating Score (four ultra-processed foods, range: 0–8). Associations were tested using linear regression (scores vs. stool consistency) and Poisson regression (scores vs. constipation), with the significance level set at p<0.05.

**Results:**

Among 394 children (mean age: 7.9 years; SD=0.4), constipation prevalence was 10.2%. Most (68.5%) had daily bowel movements, with type 3 on the Bristol Scale being the most frequent (43.8%). Mean Healthy Eating Score was 3.7; Unhealthy Eating Score was 4.0, with no significant differences by constipation. The Unhealthy Eating Score was negatively associated with stool consistency (β=-0.10; 95%CI -0.18 to -0.02), but not with constipation.

**Conclusions:**

In school-aged children, poor dietary quality, indicated by the consumption of ultraprocessed foods and sugar-sweetened beverages, was associated with harder stools, but not with constipation. These findings reinforce the usefulness of SISVAN dietary assessment forms for identifying children at risk of constipation.

## RESUMO

**Objetivo:**

Investigar a associação entre constipação intestinal e consistência das fezes com marcadores de qualidade da dieta em crianças em idade escolar.

**Métodos:**

Estudo transversal realizado com dados da segunda fase do Estudo de Coorte Infantil de Botucatu. A consistência das fezes foi avaliada pela Escala de Forma das Fezes de Bristol, e a constipação (sim/não) foi definida segundo os critérios de Roma IV adaptado. O consumo alimentar (sim/não), em dias de semana e fins de semana, de sete alimentos utilizados como marcadores de qualidade da dieta pelo Sistema de Vigilância Alimentar e Nutricional (SISVAN) foi obtido por entrevistas telefônicas. Foram calculados o Escore de Alimentação Saudável (três alimentos *in natura* ou minimamente processados; variação: 0–6) e o Escore de Alimentação Não Saudável (quatro alimentos ultraprocessados; variação: 0–8). As associações foram analisadas por regressão linear (escores *vs.* consistência das fezes) e regressão de Poisson (escores *vs.* constipação), adotando-se nível de significância de p<0,05.

**Resultados:**

Foram avaliadas 394 crianças (idade média: 7,9 anos; desvio padrão — DP=0,4), com prevalência de constipação de 10,2%. A maioria apresentou evacuações diárias (68,5%), sendo o tipo 3 o mais frequente (43,8%). O Escore Médio de Alimentação Saudável foi de 3,7 e o Escore de Alimentação Não Saudável foi de 4,0, sem diferenças segundo a presença de constipação. Observou-se associação negativa entre o Escore de Alimentação Não Saudável e a consistência das fezes (β=-0,10; IC95% -0,18 a -0,02), mas não com a constipação.

**Conclusões:**

Em crianças em idade escolar, a pior qualidade da dieta, caracterizada pelo consumo de alimentos ultraprocessados e bebidas adoçadas com açúcar, associou-se a fezes mais endurecidas, mas não à constipação intestinal. Os achados reforçam a utilidade dos marcadores alimentares do SISVAN na identificação de crianças em risco de alterações do hábito intestinal.

## INTRODUCTION

Diet is a major determinant of health throughout life, particularly during childhood, when adequate nutrition is essential for growth, development, and disease prevention. However, global shifts in dietary patterns, marked by increased ultra-processed food consumption and reduced intake of fruits, vegetables, legumes, and fiber, have raised concerns, contributing to diseases including functional gastrointestinal disorders (FGIDs).^1,2^

Among FGIDs, intestinal constipation stands out as a common condition in pediatrics, characterized by painful and infrequent bowel movements and a multifactorial etiology.^3^Early diagnosis is essential to prevent progression to more severe and difficult-to-treat cases, and current diagnostic criteria aim to identify constipation in its early stages.^4^ Diagnosis is currently based on the Rome IV criteria.^3^

A systematic review and meta-analysis evaluating the global prevalence of constipation according to the Rome I, II, III, and IV diagnostic criteria found prevalences of 15.3, 11.2, 10.4, and 10.1%, respectively,^5^ highlighting constipation as a public health concern worldwide. Focusing on children, a review of 14 studies conducted in Europe, North America, and Latin America reported a 12% prevalence of functional constipation, the most frequently diagnosed disorder when assessed with the Pediatric Gastrointestinal Symptom Questionnaire (QPGS-RIV), and an overall prevalence of FGID of 23%, with the highest rates observed in the Americas (23.67%).^6^

Although the Rome IV criteria consider hard stool consistency in diagnosing constipation, a more detailed assessment of stool form and consistency may aid in the early detection of bowel habit alterations. In this context, the Bristol Stool Form Scale, which classifies stools into seven types, from the hardest (Type 1) to the softest (Type 7),^7^ is widely used in clinical practice to improve stool characterization and symptom correlation. The scale has been validated for the Brazilian population^8^ and is frequently used in population-based studies, showing significant differences in stool types between children with and without constipation. For instance, Andreoli et al. reported that 40.8% of children classified as constipated by Rome IV criteria had Type 1 or 2 stools on the Bristol Scale.^9^

The relationship between dietary consumption and constipation has been widely studied in recent decades; however, the available evidence remains inconsistent.^10^ Studies assessing food groups or dietary patterns instead of isolated nutrients have identified associations with functional constipation. In Brazil, Andreoli et al. found a higher prevalence of constipation in children who did not consume fruits and vegetables, regularly consumed fried foods and chocolate-flavored milk drinks, and drank less than 600 mL of water daily.^9^ Similar findings have been reported in other countries. In Japan, greater fruit and vegetable consumption was associated with a lower prevalence of constipation among children and adolescents aged two to 18 years.^11^ In India, children aged two to 12 years with higher fruit and vegetable consumption also showed a lower frequency of constipation.^12^

Further investigation into the relationship between current dietary patterns and the prevalence of constipation and stool consistency changes in the pediatric population is crucial, particularly in Brazil, where a pronounced nutritional transition and rising childhood overweight rates reflect changing eating habits.^13^ Thus, this study aimed to investigate the association between dietary consumption and bowel habits, specifically intestinal constipation and stool consistency, among school-aged children.

## METHOD

This is a cross-sectional study nested within the second phase of the Cohort of Infants from Botucatu (ClaB) study, a population-based prospective cohort that initially included 650 mothers and 656 live-born infants between June 29, 2015, and January 11, 2016. During the first year of life, children were assessed for growth, development, morbidity, feeding practices, and healthcare use. Details on cohort recruitment are available elsewhere.^14^

**Figure 1:**
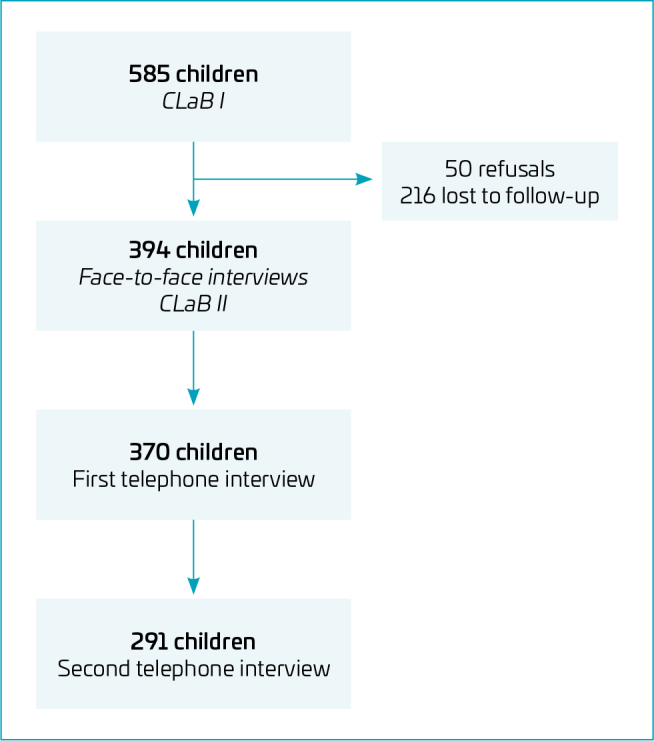
Flow diagram of data collection in the Botucatu Infant Cohort Study (CLaB Study), Phase II, Botucatu, SP, Brazil, 2023–2024.

The second phase of the CLaB study was conducted when the children were between seven and eight years old, comprising the eligible population for the present study. To locate these children, caregivers were contacted by phone or in person using the addresses provided during phase I; when unsuccessful, letters were sent, and local health units and schools were contacted. A total of 394 children were successfully located and included in phase II of the CLaB study (Figure 1).

This study followed the Strengthening the Reporting of Observational Studies in Epidemiology (STROBE) Statement and was approved by the Research Ethics Committee of Botucatu Medical School — São Paulo State University — UNESP (Certificate of Presentation for Ethical Appreciation — CAAE: 57893322.7.0000.5411). All participating families signed a new informed consent form for phase II.

Data were collected between April 2023 and October 2024 in three stages:

1. A home interview was conducted to collect sociodemographic, morbidity, behavioral, and anthropometric data, along with an assessment of the children’s current nutritional status according to Brazilian Ministry of Health criteria.^15^
2 and 3. Two telephone interviews focused on dietary consumption.

Further details on the second phase of data collection can be found in a previous publication.^16^

Diet quality was assessed using the Food and Nutrition Surveillance System (SISVAN) Food Consumption Screeners questionnaire for children aged two years and older.^17^ Dietary data were collected through two telephone interviews: one on a weekday and one on a weekend day, given potential variation in weekend consumption. The questions addressed consumption on the previous day of seven food items/food groups: beans; fresh fruits (excluding juice); vegetables (excluding potatoes, cassava, manioc, taro, and yam); hamburgers and processed meats (ham, bologna, salami, sausage, hot dogs); sweetened beverages (soda, boxed juice, powdered drinks, syrups, sugar-sweetened juices); instant noodles, packaged snacks, and savory crackers; and filled cookies, sweets, and candies.^17^ These screeners have been validated as indicators of diet quality^18^ and are recommended by the Brazilian Ministry of Health for use in primary healthcare settings.

To summarize diet quality, two scores were calculated: the Healthy Consumption Score (HCS), based on the sum of affirmative responses for fresh fruits, beans, and vegetables, and the Unhealthy Consumption Score (UCS), based on the sum of affirmative responses for instant noodles, sweetened beverages, processed meats, and sweets. Scores combined data for both interview days (one weekday and one weekend/holiday). For each dietary screener, consumption on both days scored 2 points, on one day 1 point, and on neither day 0 points. The HCS ranged from 0–6, and the UCS from 0–8. The scoring criteria were based on Louzada et al., adapted to incorporate data from two interview days.^18^

To assess functional constipation, Rome IV-based questions for children over four years of age^3^ selected from the Pediatric Functional Constipation Questionnaire — Parent Report (PedFCQuest-PR)^19^ were applied during the in-person interview. The questions included:

1. How often does the child have a bowel movement in the toilet? (every day, three to four times a week, once or twice a week, less than once a week);
2. Are the child’s stools hard? (never, occasionally, frequently, always);
3. Does the child feel pain when passing stool? (no pain, slight pain, moderate pain, severe pain);
4. Do the child’s stools clog the toilet? (never, occasionally, frequently, always);
5. Does the child soil their clothes with stool? (never, occasionally, frequently, always).

Children were considered constipated if, in the last four weeks, they met at least two of the following criteria:

1. Two or fewer bowel movements per week;
2. Hard stools, frequently or always;
3. Moderate or severe pain during defecation;
4. Large diameter stools that frequently or always clog the toilet;
5. The child frequently or always soils their clothes with stool.^3^

Rome IV criteria also include the presence of retentive posturing and a large fecal mass in the rectum. However, these indicators were not assessed because they require clinical evaluation and are difficult for caregivers to observe.^20^ The inclusion of fecal soiling reflects its relevance in identifying chronic functional constipation, as it is easily recognized by caregivers and commonly associated with stool retention and overflow incontinence.^21^ Therefore, constipation assessment in this study was based on adapted Rome IV criteria.

Stool consistency was evaluated using the Bristol Stool Form Scale.^8^ During the in-person interview, caregivers were shown a visual chart depicting the seven stool types, ranging from type 1 (separate hard lumps) to type 7 (watery, no solid pieces). Responses were recorded as a continuous variable (1–7), without predefined cutoffs.

Data were collected using paper-based forms and later entered into a structured Lime Survey database. Results were exported to Microsoft Excel, and data consistency was routinely verified.

Descriptive analyses were performed for all variables. Categorical variables were summarized using absolute and relative frequencies, and continuous variables were described as means and standard deviations, for the total sample and stratified by constipation status.

All analyses compared children with and without constipation. Categorical variables were assessed using the chi-square test, while differences in Bristol Stool Form Scale scores (1–7) were evaluated with the Mann-Whitney test. Previous-day food consumption, on weekdays and weekends, was compared between groups using the chi-square test for proportions.

Associations between HCS (0–6), UCS (0–8), and stool consistency (BSFS score 1–7) were analyzed using linear regression models. Associations between the HCS and UCS with constipation were assessed using Poisson regression models with robust variance estimation. Prevalence ratios (PRs) and their corresponding 95% confidence intervals (CIs) were reported. No additional subgroup or sensitivity analyses were performed.

All analyses were performed using the Statistical Package for the Social Sciences (SPSS) version 29.0 and the Statistical Analysis System (SAS), with a significance level set at p<0.05.

## RESULTS

The mean age of the 394 children included in the study was 7.9 years (range: 6.9–9.0 years; standard deviation=0.4). The majority were male (55.3%), white-skinned (67.3%), and classified as eutrophic (57.1%) based on body mass index for age (BMI-for-age) z-scores. Notably, nearly 40% of the children were classified as overweight. Most mothers were employed outside the home (65.8%), did not participate in cash transfer programs (73.0%), had a partner (69.0%), and had completed between nine and 11 years of schooling (54.0%), corresponding to at least some secondary education (Table 1).

**Table 1:** Characteristics of children and mothers participating in the study. CLaB Phase II Study, Botucatu, SP, Brazil, 2023–2024 (n=394).

Characteristics^*^	Total^*^ n (%)	Constipation		p-value
		Yes	No	
		n (%)	n (%)	
Child characteristics				
Infant sex				
Male	218 (55.3)	18 (45.0)	200 (56.6)	0.166
Female	176 (44.7)	22 (55.0)	154 (43.5)	
Skin color				
White	265 (67.3)	20 (50.0)^a^	245 (69.2)^a^	
Pardo	107 (27.2)	20 (50.0)^a^	87 (24.6)^b^	0.001
Black	22 (5.6)	0 (0)^b^	22 (6.2)^c^	
Nutritional status^*^				
Normal weight	222 (57.1)	21 (55.3)	201 (57.3)	
Underweight	14 (3.6)	2 (5.3)	12 (3.4)	0.932
Overweight	77 (19.8)	7 (18.4)	70 (19.9)	
Obesity	76 (19.5)	8 (21.1)	68 (19.4)	
Mothercharacteristics				
Workers outside the home^*^				
Yes	258 (65.8)	30 (75.0)	228 (64.8)	0.196
No	134 (34.2)	10 (25.0)	124 (35.2)	
Participate in Cash transfer programs^*^				
Yes	106 (27.0)	14 (35.0)	92 (26.1)	0.232
No	286 (73.0)	26 (65.0)	260 (73.9)	
Has a partner				
Yes	272 (69.0)	24 (60.0)	248 (70.1)	0.192
No	122 (31.0)	16 (40.0)	106 (29.9)	
Schooling (years)^*^				
≥12	125 (32.0)	11 (27.5)	114 (32.5)	0.267
9 to 11	211 (54.0)	20 (50.0)	191 (54.4)	
≤8	55 (14.1)	9 (22.5)	46 (13.1)	

*Totals differ due to item non-response; analyses based on available cases (nutritional status, workers outside the home, participate in cash transfer programs and schooling)

Note: Frequencies with the same letter in the column are not statistically different according to the proportion comparison test analogous to the χ^2^ test.

Based on the combination of signs and symptoms, 10.2% of the children were classified as having constipation. When comparing characteristics between constipated and non-constipated children, the only significant difference was observed for skin color (Table 1). Constipation prevalence was 7.5% among white children and 18.7% among brown children, more than twice as high. Among constipated children, half were White, and half were Brown, whereas most non-constipated children were White (69.2%).

Table 2 presents the findings regarding bowel habits among the children evaluated, as well as comparisons between children with and without constipation. Most children had daily bowel movements (68.5%), did not have hard stools (60.9%), did not experience pain during defecation (74.6%), never clogged the toilet (78.7%), and never soiled their clothes with stools (70.3%).

**Table 2:** Bowel habits for the assessment of constipation in school-aged children. CLaB Study Phase II, Botucatu, SP, Brazil, 2023–2024 (n=394).

Bowel habits	Total n (%)	Constipation		p-value
		Yes	No	
		n (%)	n (%)	
What is the frequency of the child’s bowel movements?				
Less than once a week	3 (0.8)	2 (5.0)^a^	1 (0.03)^a^	<0.001
Daily	270 (68.5)	14 (35.0)^b^	256 (72.3)^b^	
Three to four times a week	98 (24.9)	12 (30.0)^b^	86 (24.3)^c^	
One or twice a week	23 (5.8)	12 (30.0)^b^	11 (3.1)^d^	
Does the child have pain when having a bowel movement?				
Severe pain	15 (3.8)	10 (25.0)^a^	5 (1.4)^a^	<0.001
Moderate pain	38 (9.6)	15 (37.5)^a^	23 (6.5)^b^	
Mild pain	47 (11.9)	4 (10.0)^a^	43 (12.1)^c^	
No pain	294 (74.6)	11 (27.5)^a^	283 (79.9)^d^	
Does the stool clog the toilet?				
Always	3 (0.8)	7 (17.5)^a^	0 (0.0)^a^	<0.001
Frequently	10 (2.5)	23 (57.5)^b^	3 (0.08)^a^	
Occasionally	71 (18.0)	7 (17.5)^a^	64 (18.1)^b^	
Never	310 (78.7)	23 (57.5)^b^	287 (81.1)^c^	
Does the child soil their clothes with stool?				
Always	12 (3.0)	4 (10.0)^a^	8 (2.3)^a^	<0.001
Frequently	15 (3.8)	6 (15.0)^a^	9 (2.5)^a^	
Occasionally	90 (22.8)	11 (27.5)^a^	79 (22.3)^b^	
Never	277 (70.3)	19 (47.5)^b^	258 (72.9)^c^	

*χ^2^ test

Note: Frequencies with the same letter in the column are not statistically different according to the proportion comparison test analogous to the χ^2^test.

Statistically significant differences were found in all bowel habit variables when comparing children classified as constipated and those without constipation. Constipated children had less frequent bowel movements, more frequent toilet clogging, and a greater occurrence of fecal soiling (p<0.001). Nearly one-third (30.0%) had bowel movements only once or twice a week, and one-quarter experienced severe pain during defecation.

**Figure 2:**
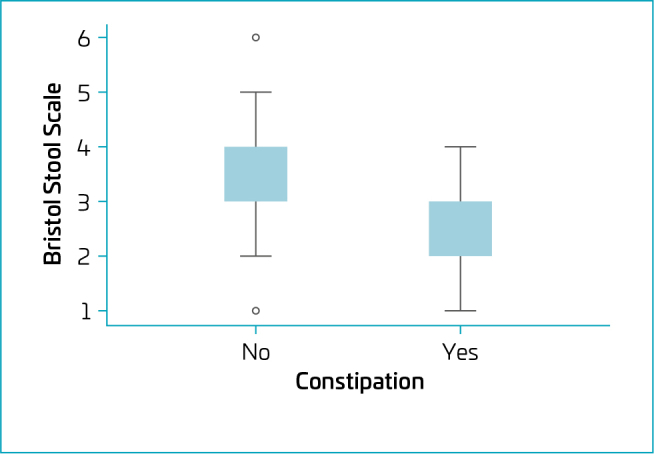
Evaluation of the Bristol stool form scale as a score (1 to 7) with or without constipation; note that a lower score indicates greater stool consistency. CLaB Study Phase II, Botucatu, SP, Brazil, 2023–2024 (n=394)^*^.

Considering the Bristol Stool Form Scale assessed as a numerical score (ranging from 1 to 7), the median score among the 379 children with available data was 3.0 (interquartile range [IQR] 3.0–4.0). Among non-constipated children, the median was also 3.0 (IQR 3.0–4.0), while among constipated children, the median was 3.0 (IQR 2.0–3.0), with a statistically significant difference between the groups (p=0.004) (Figure 2).

*Mann-Whitney test.

Table 3 presents the consumption of dietary screeners on weekdays and weekends for the total sample of children and according to the presence of constipation. On weekdays, the most frequently consumed foods were beans (76.0%), sweetened beverages (68.8%), and fruits (65.5%). On weekends, sweetened beverages were the most consumed item (84.0%), followed by fruits (59.3%) and sweets (57.2%). The frequency of beans (p<0.001) and vegetable (p=0.001) consumption was significantly higher on weekdays (Monday to Friday) compared to weekends/holidays, whereas the consumption of sweetened beverages was significantly higher on weekends (p<0.001). When comparing dietary screener consumption between children with and without constipation, a significantly higher consumption of sweetened beverages was observed among constipated children during weekends. However, for the other screeners, both on weekdays and weekends, no statistically significant differences were found between the groups.

**Table 3:** Consumption of Food and Nutritional Surveillance System (SISVAN) dietary screeners on weekdays and weekends in children with and without constipation. CLaB Study Phase II, Botucatu, SP, Brazil, 2023–2024.

Dietary consumption screeners from the previous day	Total			During the week			Weekends/holidays		
	During the week^*^	Weekends/holidays^*^	p-value	Constipated	Not constipated	p-value	Constipated	Not constipated	p-value
	n (%)	n (%)		n (%)	n (%)		n (%)	n (%)	
Beans									
Yes	275 (76.0)	159 (54.8)		27 (71.1)	248 (76.5)		16 (57.1)	143 (54.6)	
No	81 (22.4)	131 (45.2)	<0.001	9 (23.7)	72 (22.2)	0.174	12 (42.9)	119 (45.4)	0.795
I don’t know	6 (1.7)	0 (0.0)		2 (5.3)	4 (1.2)		0 (0.0)	0 (0.0)	
Fresh fruits									
Yes	237 (65.5)	172 (59.3)		25 (65.8)	212 (65.4)		17 (60.7)	155 (59.2)	
No	109 (30.1)	114 (39.3)	0.007	11 (28.9)	98 (30.2)	0.957	10 (35.7)	104 (39.7)	0.551
I don’t know	16 (4.4)	4 (1.4)		2 (5.3)	14 (4.3)		1 (3.6)	3 (1.1)	
Vegetables (greens and legumes)									
Yes	211 (58.3)	128 (44.1)		26 (68.4)	185 (57.1)		11 (39.3)	117 (44.7)	
No	132 (36.5)	159 (54.8)	<0.001	11 (28.9)	121 (37.3)	0.375	17 (60.7)	142 (54.2)	0.711
I don’t know	19 (5.2)	3 (1.0)		1 (2.6)	18 (5.6)		0 (0.0)	3 (1.1)	
Hamburgers a/or processed meats									
Yes	127 (35.1)	132 (45.5)		11 (28.9)	116 (35.8)		14 (50.0)	118 (45.0)	
No	227 (62.7)	157 (54.1)	0.001	26 (68.4)	201 (62.0)	0.701	14 (50.0)	143 (54.6)	0.843
I don’t know	8 (2.2)	1 (0.3)		1 (2.6)	7 (2.2)		0 (0.0)	1 (0.4)	
Sugary drinks									
Yes	249 (68.8)	245 (84.5)		27 (71.1)	222 (68.5)		26 (92.9)	219 (83.6)	
No	107 (29.6)	43 (14.8)	<0.001	9 (23.7)	98 (30.2)	0.145	1 (3.6)	42 (16.0)	0.036
I don’t know	6 (1.7)	2 (0.7)		2 (5.3)	4 (1.2)		1 (3.6)	1 (0.4)	
Instant noodles, packaged snacks, or salty biscuits									
Yes	101 (27.9)	84 (29.0)		15 (39.5)	86 (26.5)		9 (32.1)	75 (28.6)	
No	253 (69.9)	204 (70.3)	0.287	22 (57.9)	231 (71.3)	0.229	18 (64.3)	186 (71.0)	0.135
I don’t know	8 (2.2)	2 (0.7)		1 (2.6)	7 (2.2)		1 (3.6)	1 (0.4)	
Filled cookies, sweets, or candies									
Yes	191 (52.8)	166 (57.2)		20 (52.6)	171 (52.8)		18 (64.3)	148 (56.5)	
No	162 (44.8)	123 (42.4)	0.061	17 (44.7)	145 (44.8)	0.998	10 (35.7)	113 (43.1)	0.704
I don’t know	9 (2.5)	1 (0.3)		1 (2.6)	8 (2.5)		0 (0.0)	1 (0.4)	

*χ^2^ test for difference in proportions.

The mean Healthy Consumption Score among all children was 3.7±1.45, with scores of 3.8±1.50 for children with constipation and 3.7±1.44 for those without constipation. The mean Unhealthy Consumption Score was 4.5±1.64 for constipated children and 4.0±1.65 for non-constipated children.

Table 4 presents an inverse association between the Unhealthy Consumption Score, an indicator of poorer diet quality, and the Bristol Stool Form Scale score, which reflects stool consistency (β=-0.10; 95% confidence interval — 95%CI -0.18 to -0.02). Statistical analysis indicates that each additional point in the Unhealthy Consumption Score corresponds to a 0.10-point decrease in the Bristol Stool Form Scale score. Furthermore, no significant association was found between either the Healthy or Unhealthy Consumption Scores and the presence of constipation.

**Table 4:** Results of the analyses examining the association between healthy and unhealthy dietary consumption scores, Bristol stool consistency scores, and the presence of constipation. CLaB Study Phase II, Botucatu, SP, Brazil, 2023–2024.

Bristol stool scale	β^*^(95%CI)	p-value
Healthy score	-0,01 (-0,10; -0,09)	**0,893**
Unhealthy score	-0,10 (-0,18; -0,02)	**0,013**
**Constipation**	**PR^†^ (95%CI)**	**p-value**
Healthy score	1,06 (0,80; 1,41)	0,675
Unhealthy score	1,17 (0,93; 1,47)	0,188

*Linear regression;

^†^Poisson regression with robust variance. CI: confidence interval; PR: prevalence ratio.

## DISCUSSION

This study showed that poorer diet quality was associated with harder stools, as assessed by the Bristol Stool Form Scale, but not with clinically diagnosed constipation. Constipation affected 10.2% of the children, with notable differences in stool consistency and bowel habits between those with and without the condition. These results highlight the need to consider both stool consistency and clinical criteria when assessing intestinal health, as constipation can impair quality of life and have short-and long-term consequences.^22^

The prevalence of constipation found is similar to that reported in other pediatric studies, such as the 12% prevalence reported in a review study investigating this condition among children and adolescents aged four to 18 years,^6^ and among Japanese children aged five to six years (8.4%).^11^ A national study in Minas Gerais, involving 152 children aged four to seven years, reported a higher prevalence of 32.7%, possibly reflecting the Botucatu cohort’s more favorable socioeconomic profile, including higher maternal education.^23^ Constipation has been reported as more prevalent in lower-income contexts, often associated with poor dietary habits.^24,25^ Additionally, in this study, a higher prevalence of constipation was observed among children of mixed race, corroborating findings from previous studies, including those involving adults.^26^ Because adapted Rome IV criteria were used, comparisons with studies applying the full criteria require caution.

Although no association between dietary consumption screeners from SISVAN and the presence of constipation was confirmed, an inverse relationship was observed between the Unhealthy Eating Score and stool consistency, with higher consumption of these foods associated with harder stools. Weekdayweekend variation showed greater minimally processed food intake during the week and higher ultra-processed consumption on weekends, patterns linked to poorer diet quality and chronic disease risk.^27,28^ This difference may relate to family routines and even school meal practices, as weekday meals tend to be more balanced, whereas weekends favor convenience.^29^Sugar-sweetened industrialized beverages were consumed more frequently by constipated children on weekends, representing a potential intervention target. Although excessive consumption of sugary drinks may contribute to weight gain, it does not appear to facilitate softer stools, which may similarly affect children’s stool consistency.

The Bristol Stool Form Scale demonstrated a significant negative association between the Unhealthy Diet Score and Bristol Score, indicating that higher consumption of ultra-processed foods correlates with harder stools. Diets rich in sugars and saturated fats may impair stool texture, partly because ultra-processed foods are typically low in fiber, adversely affecting gut microbiota and increasing the likelihood of harder stools.^30^This reinforces the relevance of preventive strategies focused on improving diet quality.

The study has limitations. Its cross-sectional design precludes causal inference, although validated instruments strengthen the findings. Despite the limited sample size, restricted to the available CLaB population, statistically significant associations were detected, though some may have gone unnoticed. Hydration status and physical activity were not assessed and could not be controlled, potentially introducing residual confounding in the relationship between diet and stool consistency.

Conducting the study in a single medium-sized city in Brazilian city may limit generalizability; however, approximately 28% of Brazilians live in cities with 100,000–500,000 inhabitants, supporting contextual relevance. Further nationally representative studies are needed to better understand pediatric constipation prevalence and regional and social disparities.

The use of adapted Rome IV criteria is another limitation. The absence of data on retentive posturing and rectal fecal accumulation may have led to underestimation of prevalence, although estimates were close to those reported in the literature. Lack of information on laxative and antibiotic use, both influencing stool frequency and consistency, should also be acknowledged.

It is worth noting that despite a significant loss to follow-up (32.6%) compared to the initial sample of 585 children assessed at 12 months of age, no statistically significant differences were observed between the analyzed sample and the original cohort, supporting the lack of bias in the results presented here (data not shown).

Our findings suggest that weekend-focused educational interventions, reducing sugary drinks and ultra-processed foods while promoting legumes and vegetables, may be cost-effective. Integrating SISVAN dietary screeners with the Bristol Stool Form Scale enables rapid screening and real-time tracking of stool consistency, offering healthcare teams a practical target for follow-up. Such strategies may improve intestinal health and support broader public health efforts promoting healthy dietary habits early in life.

In school-aged children, poor diet quality, particularly high consumption of ultra-processed foods and sugar-sweetened beverages, was associated with harder stools, although not with functional constipation. These findings highlight the value of SISVAN dietary screeners for identifying dietary patterns associated with harder stools and the potential risk of constipation.

## Data Availability

The database that originated the article is available with the corresponding author.

## References

[1] Batista M, Rissin A (2003). A transição nutricional no Brasil: tendências regionais e temporais.. Cad Saúde Pública..

[2] Moraes ES, Fernandez JQ, Detregiachi CR (2023). Determinantes das escolhas alimentares de nutricionistas sob a ótica da área de atuação.. Rev Contemp.

[3] Hyams JS, Di Lorenzo C, Saps M, Shulman RJ, Staiano A, van Tilburg M (2016). Childhood functional gastrointestinal disorders: child/adolescent.. Gastroenterology.

[4] Maffei HV, Morais MB (2018). Proposals to approximate the pediatric Rome constipation criteria to everyday practice.. Arq Gastroenterol.

[5] Barberio B, Judge C, Savarino EV, Ford AC (2021). Global prevalence of functional constipation according to the Rome criteria: a systematic review and meta-analysis.. Lancet Gastroenterol Hepatol.

[6] Velasco-Benítez CA, Collazo-Saa LI, Garcàa-Perdomo HA (2022). A systematic review and meta-analysis in schoolchildren and adolescents with functional gastrointestinal disorders according to Rome IV criteria.. Arq Gastroenterol.

[7] Lewis SJ, Heaton KW (1997). Stool form scale as a useful guide to intestinal transit time.. Scand J Gastroenterol.

[8] Martinez AP, Azevedo GR (2012). The Bristol Stool Form Scale: its translation to Portuguese, cultural adaptation and validation.. Rev Latino-Am Enfermagem.

[9] Andreoli CS, Ribeiro-Vieira SA, Fonsêca PC, Moreira AV, Ribeiro SM, Franceschini SC (2018). Markers of healthy eating habits, water intake, and constipation in children between 4 and 7 years of age.. Rev Nutr.

[10] Mello PP, Eifer DA, Mello ED (2018). Use of fibers in childhood constipation treatment: systematic review with meta-analysis.. J Pediatr (Rio J).

[11] Asakura K, Masayasu S, Sasaki S (2017). Dietary intake, physical activity, and time management are associated with constipation in preschool children in Japan.. Asia Pac J Clin Nutr.

[12] Sujatha B, Velayutham DR, Deivamani N, Bavanandam S (2015). Normal bowel pattern in children and dietary and other precipitating factors in functional constipation.. J Clin Diagn Res.

[13] Brasil. (2019). Ministério da Saúde [homepage on the Internet]. Atlas da obesidade infantil no Brasil. Brasília (DF): Ministério da Saúde;. http://189.28.128.100/dab/docs/portaldab/publicacoes/dados_atlas_obesidade.pdf.

[14] Almeida MA, Rossato SL, Ferrari AP, Gomes CB, Tonete VL, Parada CM (2022). The determinants of complementary feeding introduction vary according to the type of food and infants’ ages: a cohort study, ClaB, Brazil.. Matern Child Health J.

[15] Brasil (2022). Ministério da Saúde [homepage on the Internet]. Guia para a organização da vigilância alimentar e nutricional na atenção primária à saúde. Brasília (DF): Ministério da Saúde;. http://bvsms.saude.gov.br/bvs/publicacoes/guia_organizacao_vigilancia_alimentar_nutricional.pdf.

[16] Gomes CB, Parada CM, Parenti AB, Spadotto GC, Alves MS, Padovani FH (2025). Effects of gestational weight gain on emotional and behavioral problems in children: results from the CLaB study.. PLoS One.

[17] Brasil (2015). Ministério da Saúde [homepage on the Internet]. Orientações para avaliação de marcadores de consumo alimentar na atenção básica. Brasília (DF): Ministério da Saúde;. https://bvsms.saude.gov.br/bvs/publicacoes/marcadores_consumo_alimentar_atencao_basica.pdf.

[18] Louzada ML, Couto VD, Rauber F, Tramontt CR, Santos TS, Lourenço BH (2023). Marcadores do Sistema de Vigilância Alimentar e Nutricional predizem qualidade da dieta.. Rev Saude Publica.

[19] Gamarra AC, Carvalho MA, Machado NC (2022). Pediatric Functional Constipation Questionnaire–Parent Report (PedFCQuest-PR): development and validation.. J Pediatr (Rio J).

[20] Hyman PE, Milla PJ, Benninga MA, Davidson GP, Fleisher DF, Taminiau J (2006). Childhood functional gastrointestinal disorders: neonate/toddler.. Gastroenterology.

[21] Junqueira JC, Calçado AC, Gracia J, Guerra SP, Carvalho SR, Valladares MA (2009). Constipação intestinal crônica na criança e no adolescente. São Paulo:. Nestle Nutrition Institute.

[22] Belsey J, Greenfield S, Candy D, Geraint M (2010). Systematic review: impact of constipation on quality of life in adults and children.. Aliment Pharmacol Ther.

[23] Viola PC, Ribeiro SA, Carvalho RR, Andreoli CS, Novaes JF, Priore SE (2023). Situação socioeconômica, tempo de tela e permanência na escola e consumo alimentar de crianças.. Ciênc Saúde Colet.

[24] Kawaguti FS, Klug WA, Fang CB, Ortiz JA, Capelhucnick  P (2008). Constipação na gravidez.. Rev Bras Coloproctol.

[25] Rodrigues VM, Fiates GM (2012). Hábitos alimentares e comportamento de consumo infantil: influência da renda familiar e do hábito de assistir à televisão.. Rev Nutr.

[26] Collete VL, Araújo CL, Madruga SW (2010). Prevalência e fatores associados à constipação intestinal: um estudo de base populacional em Pelotas, Rio Grande do Sul, Brasil, 2007.. Cad Saúde Pública..

[27] Khandpur N, Neri DA, Monteiro C, Mazur A, Frelut ML, Boyland E (2020). Ultra-processed food consumption among the paediatric population: an overview and call to action from the European Childhood Obesity Group.. Ann Nutr Metab.

[28] Mescoloto SB, Pongiluppi G, Domene SM (2024). Ultra-processed food consumption and children and adolescents’ health.. J Pediatr (Rio J).

[29] Monteiro LS, Hassan BK, Estima CC, Souza AM, Verly E, Sichieri R (2017). Consumo alimentar segundo os dias da semana: Inquérito Nacional de Alimentação, 2008-2009.. Rev Saude Publica.

[30] Agostinho MR (2016). Valente A [homepage on the Internet]. Ingestão de fibra alimentar e prevalência de obstipação em crianças dos 2-11 anos. Lisboa: Atlântica Repositório Científico;. http://hdl.handle.net/10884/980.

